# Serological and Antibiotic Resistance Patterns As Well As Molecular Characterization of *Vibrio parahaemolyticus* Isolated from Coastal Waters in the Eastern Province of Saudi Arabia

**DOI:** 10.1007/s44197-022-00071-3

**Published:** 2022-10-14

**Authors:** Nasreldin Elhadi, Lamya Zohair Yamani, Mohammed Aljeldah, Amer Ibrahim Alomar, Hafiz Ibrahim, Asim Diab

**Affiliations:** 1 Department of Clinical Laboratory Science, College of Applied Medical Sciences, Imam Abdulrahman Bin Faisal University, P.O. Box 2435, Dammam, 31441 Kingdom of Saudi Arabia; 2 Department of Clinical Laboratory Sciences, College of Applied Medical Sciences, University of Hafr Al-Batin, Hafr-Al Batin, Kingdom of Saudi Arabia; 3 Department of Microbiology, College of Medicine, Imam Abdulrahman Bin Faisal University, Dammam, Kingdom of Saudi Arabia

**Keywords:** Coastal water, *Vibrio parahaemolyticus*, Serotypes, Antimicrobial resistance, AP-PCR

## Abstract

*Vibrio parahaemolyticus* belongs to the halophilic genus of *Vibrionaceae* family that inhabits coastal and marine environments and is a major food-borne pathogen. In the Gulf Cooperation Council (GCC) countries and Saudi Arabia in particular, there is a lack of information regarding the detection of pandemic clone or serovariants of *V. parahaemolyticus* pandemic clones. Here, 400 seawater samples were collected and examined for the presence of *V. parahaemolyticus* from 10 locations along the coast of Eastern Province in Saudi Arabia. The recovered isolates were serotyped, and studied for antimicrobial resistance, virulence genes, and markers of pandemicity using PCR and Arbitrarily primed (AP)-PCR typing patterns. All 40 isolates were tested negative for *tdh, trh*, and *tox*RS genes. Six serotypes were identified and three were clinically significant. Antibiotic susceptibility testing of isolates revealed high resistance towards penicillins, cephalosporins, and polymyxin; 60% of isolates were multi-drug resistant, whereas all isolates were susceptible to quinolones, carbapenems, sulfonamides, and tetracycline. The multiple antibiotic resistance (MAR) index among antibiotic resistance patterns of isolates revealed that 12 (30%) isolates had recorded significant MAR index higher than 0.2. AP-PCR fingerprinting could group all isolates into five distinct and identical pattern clusters with more than 85% similarity. Our findings demonstrate that pandemic serovariants of pandemic clones were not exclusively limited to strains isolated from fecal specimens of infected patients. Nine environmental strains of serotype O1:KUT, O1: K25, and O5:K17 were isolated from costal seawater, and thus the spread of these serovariants strains of pandemic clone of *V. parahaemolyticus* in the environment is to avoid any kind of threat to public health.

## Introduction

*Vibrio parahaemolyticus* is a medically important organism listed under the family of *Vibrionaceae* of *Vibrio* species; it lives in marine environments [1, 2]. This bacterium was reported for the first time in 1950 due to consumption of contaminated *shirasu*, which led to an outbreak of food poisoning ultimately leading to 20 deaths and 272 illnesses [3]. Among the *Vibrio* species, *V. parahaemolyticus* is a premier causative agent of foodborne illness [4]. In humans, infection with this bacterium may lead to gastroenteritis after ingestion of contaminated seafood products [5]. *V. parahaemolyticus* is spread worldwide along coastal environments and accumulates in the digestive tract of filter feeders such as molluscan bivalves [6]. For the pathogenic strains of *V. parahaemolyticus* to be considered toxigenic, they should be encoded with either thermostable direct hemolysin (TDH) genes or encoded with thermostable-related hemolysin (TRH) [7]. The clinical strains of *V. parahaemolyticus* isolated from fecal specimens nearly all harbor *trh* and/or *tdh* genes, but the detection of these genes in environmental isolates is usually rare [6, 8].

In 1996, the *V. parahaemolyticus* serotype O3:K6 emerged in Kolkata in Eastern India and the Bay of Bengal. It was responsible for an outbreak of acute gastroenteritis [9]. Several cases of O3:K6 pandemic clone strains were associated with foodborne outbreaks in subsequent years; sporadic cases have been reported in the Gulf coast and Atlantic coast of the United States as well as Southeast Asia [10, 11]. Similar cases were of a new serotype O3:K6 were reported in Europe, Africa, South America, and Mexico [12–15]. The spread of these remarkable and unique serotypes suggested that this pathogen was a pandemic clone, and thus universal public health measurements were needed especially with regard to the consumption of seafood [8]. Therefore, group-specific (GS) PCR-based method was developed targeted to *tox*RS gene. This gene encoding the transmembrane protein is a molecular marker for the detection of pandemic strains of this organism [11].

Strains of *V. parahaemolyticus* harboring virulence genes were responsible for most foodborne gastroenteritis outbreaks after consumption of contaminated seafood. They have been reported in the United States [16], many Asian countries [17], and South America [18]. There have been several studies on the prevalence and occurrence of pandemic clones of *V. parahaemolyticus* in coastal areas and cases have been reported among infected humans worldwide. However, there are few reports in Gulf cooperation council (GCC) countries. Relative to Asia and most of North America, *V. parahaemolyticus* cases are rarely associated with the consumption of infected seafood in Saudi Arabia and other Middle Eastern countries. This probably due to a lack of systems for monitoring foodborne pathogens and it might also be due to not reported cases. To the best of our knowledge, this is our first report on pandemic clones of *V. parahaemolyticus* serovariants in environmental seawater samples in the Eastern Province of Saudi Arabia.

## Materials and Methods

###  Sampling Sites 

**Fig. 1:**
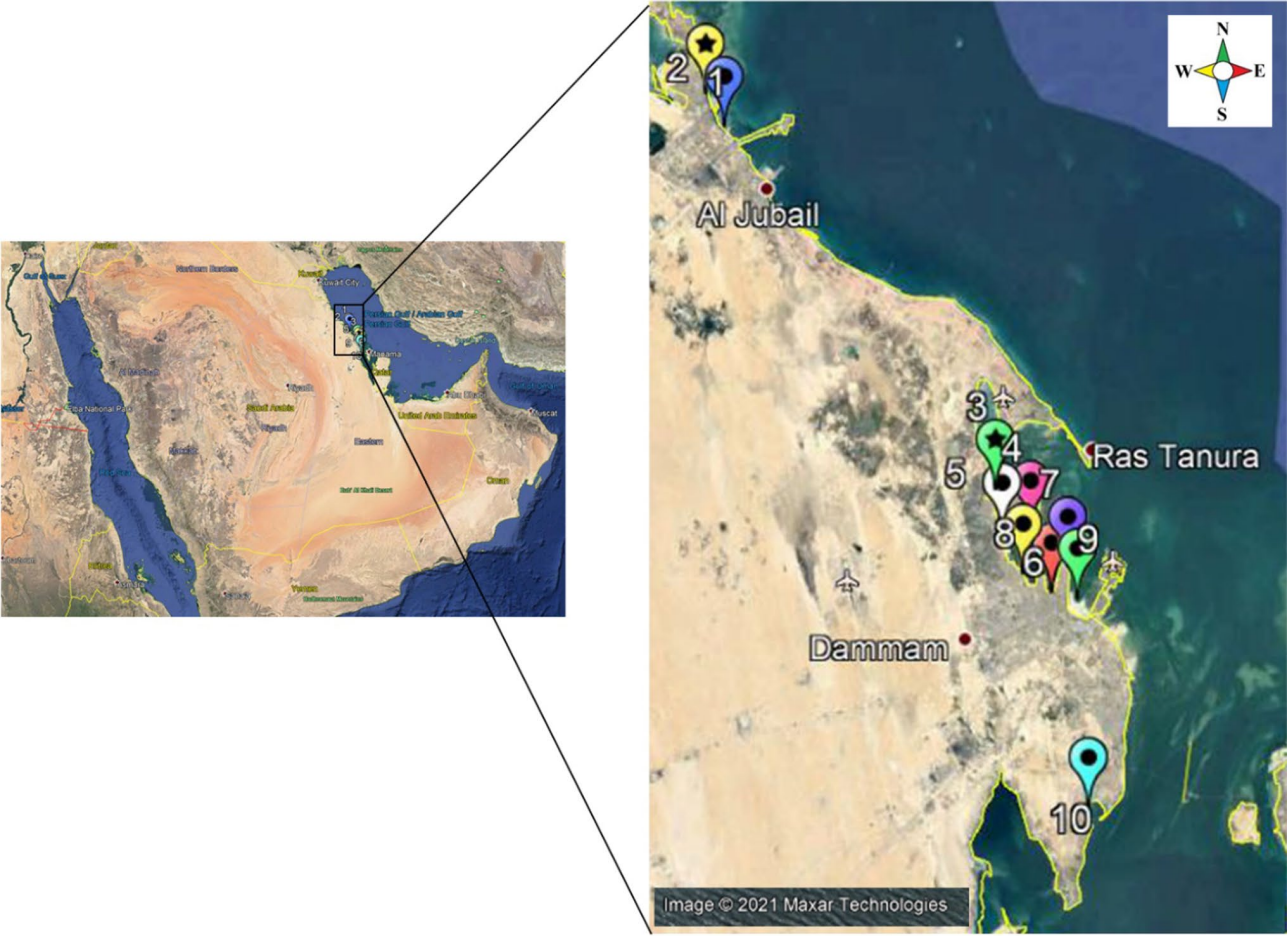
Sampling locations in Eastern Province of Saudi Arabia

The Arabian Gulf is a semi-enclosed marine that covers an area of about 240,000 km^2^. It is characterized by conspicuous fluctuations in water temperatures and high levels of salinity [19]. A significant percentage of the global sea-transported oil is shipped through the Gulf, and thus its ecosystem is under stress from continuous discharge of hydrocarbon pollutants and crude oil spills [20]. Its coastline has seen drastic economic and social development [21]. Industrial and sewage discharge combined with low water exchange rates has caused the Arabian Gulf to be one of the highest anthropogenically impacted regions in the world [21]. Ten different locations were chosen for seawater collection alongside the coastline of the Arabian Gulf. These included beaches for public use, fishing areas, and recreational water sources. The locations are Fanateer corniche (FNC), Dammam corniche (DMC), Dammam marina front (DMF), Tarout corniche (TRC), Sayhat corniche (SEC), Qatif corniche (QTC), Palm Beach Jubail (PBJ), and Almorjan Island (MOI); Fig. 1. Some locations were divided into two sites that were coordinated using Garmin GPS during sample collection (Table 1).

**Table 1:** Sampling sites

Sampling site	Site no.	Site coordination	Sampling date	Seawater parameters	
				Temperature ( °C)	Salinity (psu/ppt)
Fanateer corniche (FNC)	1	N27.12923° E049.56947°	7 April 2015	25.4	45.4
Dammam corniche (DMC)	2	N26.49129° E050.13405°	26 May 2015	29.7	44
Dammam marina front (DMF)	3	N26.19796° E050.12227°	26 May 2015	30	43.6
Tarout corniche (TRC)	4	N2654174° E050.07447°	19 October 2015	32	45.8
Sayhat corniche (SEC)	5	N26.48230° E050.06369°	19 October 2015	32.7	33.2
Qatif corniche (QTC)	6	N26.53723° E050.03073°	19 October 2015	32	29.4
Palm Beach Jubail (PBJ)	7	N27.11060° E049.57544°	28 October 2015	30.2	44.2
Almorjan Island (MOI)	8	N26.48453° E050.10513°	30 December 2015	17.4	43.6
Sayhat corniche (SEC)	9	N26.47725° E050.06583°	24 January 2016	20.7	42.3
Qatif corniche (QTC)	10	N26.55126° E050.02346°	24 January 2016	21.1	39.1

### Sample Collection and Water Parameters

The physical water parameters (temperature and salinity) were measured during samples collection via a multi-parameter water quality meter (YSI-50 series, Horiba, Japan) in accordance with the manufacturer’s instructions. Four hundred seawater samples were collected just below the surface in duplicate sterile 500 ml screw-cap bottles (Fischer, UK) from different sampling sites between April 2015 and January 2016 (Table 1). The seawater samples were collected from 10 sampling sites as illustrated in Fig. 1. The collected samples were kept in insulated coolers without ice and transferred to the laboratory within three to five hours and processed immediately upon arrival.

### Isolation and Biochemical Identification

The strains of *V. parahaemolyticus* in this study were isolated and identified according to the bacteriological analysis manual of the Food and Drug Administration [22]. All commercial culture media and reagents used in this study were purchased from Oxoid unless indicated otherwise. In accordance with the FDA method for *V. parahaemolyticus* isolation, seawater samples enriched in alkaline peptone water (APW) were adjusted to 3% sodium chloride (NaCl) concentrations [22]. The preparation of 3% NaCl-APW was performed as described in the bacteriological analytical manual [22]. Peptone and NaCl were dissolved in sterilized distilled water, dispensed into screw-cap bottles, and autoclaved. Their pH was adjusted to 8.5 ± 0.2. Following that, 25 ml of seawater samples were added to 225 ml of APW adjusted to 3% NaCl. As soon as the enrichment was completed at 35 ± 2 °C for 18–24 h, a loop full of the enriched sample in APW was streaked onto CHROMagar Vibrio (CHROM, France) and plates were incubated overnight at 37 °C. Three to five mauve color colonies on the CHROM Vibrio agar plates were then presumptively identified as *V. parahaemolyticus* and inoculated into Kliger iron medium with 1% NaCl, tryptic soy agar with 3% NaCl, and nutrient broth with 8% NaCl. Isolates positive for oxidase tests with alkaline slant and acid butt reactions in the Kliger iron agar medium were further analyzed. In nutrient broth, no growth without and growth with 8% NaCl were identified as *V. parahaemolyticus* as described elsewhere [23]. Further confirmation of *V. parahaemolyticus* used API 20E strips (Biomeruex, France). Prior to performing the API test, an inoculum of 2% saline was prepared from tryptic soy agar (TSA) colonies. ATCC 17,802 strain of *V. parahaemolyticus* was used as a positive control. Colonies of *V. parahaemolyticus* were identified presumptively and were inoculated into tryptic soy broth (TSB) supplemented with 3% sodium chloride and stored with 30% glycerol at – 80 °C.

### Nucleic Acid Extraction

The presumptive colonies of *V. parahaemolyticus* strains were sub-cultured into Luria–Bertani (LB) agar plates adjusted to 1% NaCl and incubated overnight at 37 °C. At least five colonies were boiled in 500 µl of sterile distilled water for 15 min to extract the nucleic acid [24].

### Confirmation of *V. parahaemolyticus *to Species Level Using PCR Targeted to the *tox*R Gene

All of the presumptively identified isolates of *V. parahaemolyticus* isolated via CHROMagar Vibrio and biochemical tests were further confirmed using PCR targeted to the *tox*R gene as described elsewhere [25]. The PCR amplification and primers are presented in Table 2. The reaction mixture (final volume, 20 µl) consisted of 1.2 µl of the solution containing a template of crude DNA lysate, 2 µl of 10X reaction buffer (Promega, USA), 1.6 µl of 25 mM MgCl_2_, 1.6 µl of 2.5 mM deoxynucleoside triphosphates, 0.1 µl of *Taq* polymerase (5 U/µl), 0.8 µl of each primer, and 11.9 µl of distilled water. The reactions were performed with T100 thermocycler (Biorad, USA). The *V. parahaemolyticus* strain ATCC 17,802 was included for each PCR as a positive control.

**Table 2:** Primers and amplification conditions used in this study

Gene	Primer pair	Oligonucleotide	Amplicon size (bp)	Amplification conditions	Reference
*tox*R	toxR-4 toxR-7	5’-GTCTTCTGACGCAATCGTTG-3’ 5’-ATACGAGTGGTTGCTGTCATG-3’	368	94 °C–1 min 63 °C–1.5 min 72 °C–1.5 min	[25]
*tdh*	VP-D2 VP-D1	5’-CCACTACCACTCTCATATGC-3’ 5’-GGTACTAAATGGCTGACATC-3’	251	94 °C–1 min 55 °C–1 min 72 °C–1 min	[26]
*trh*	Trh-R2 Trh-R6	5’-GGCTCAAAATGGTTAAGCG-3’ 5’-CATTTCCGCTCTCATATGC-3’	250	94 °C–1 min 55 °C–1 min 72 °C–1 min	[26]
GS-PCR	GS-VP.1 GS-VP.2	5’-TAATGAGGTAGAAACA-3’ 5’-ACGTAACGGGCCTACA-3’	651	96 °C–1 min 45 °C–2 min 72 °C–3 min	[11]

### Detection of Virulence Genes

The 40 isolates that were *tox*R positive were further amplified using *tdh* and *trh* gene primer sets [26] as described in Table 2. These primer sets produced 251 and 250-bp amplicons, respectively. The reaction mixtures (final volume, 20 µl) contained 1.2 µl of the solution containing DNA, 2 µl of 10 × reaction buffer (Promega, USA), 1.6 µl of 25 mM MgCl_2_, 0.1 µl of *Taq* polymerase (5 U/µl), 1.6 µl of 2.5 mM deoxynucleoside triphosphates solution, 0.8 µl of each primer, and 11.9 µl of distilled water. The reactions were performed with T100 thermocycler (Biorad, USA) as follows: 5 min of initial denaturation at 96 °C, 35 cycles at 94 °C for 1 min, annealing at 55 °C for 1 min, and extension and final extension at 72 °C for 1 min and 7 min, respectively. Positive DNA controls of *V. parahaemolyticus* AQ3815 and AQ 4037 for tdh and trh, respectively, were included in all PCR assays.

### Group-Specific (GS) PCR

A GS-PCR method to specifically detect the *tox*RS sequence of the new O3:K6 clone of pandemic *V. parahaemolyticus* strains was performed as described [11]; see Table 2. The reaction mixtures (final volume, 20 µl) contained 2.5 µl of the solution containing DNA (supernatant of the boiled culture diluted 1:10), 2 µl of 10 × reaction buffer (Promega, USA), 1.2 µl of 25 mM MgCl_2_, 0.1 µl of *Taq* polymerase (5 U/µl), 1 µl of 2.5 mM deoxynucleoside triphosphates solution, 2 µl of each primer solution, and 9.2 µl of distilled water. The reactions were performed with T100 thermocycler (Biorad, USA) as follows: 5 min of initial denaturation at 96 °C, 25 cycles of denaturation: 96 °C for 1 min, annealing: 45 °C for 2 min, and extension: 72 °C for 3 min and a final extension: 72 °C for 7 min. Positive DNA control of *V. parahaemolyticus* O3:K6 (VP81-Japan) was included in all PCR assays.

### Serotyping of *V. parahemolyticus*

Serotyping of all 40 isolates of *V. parahaemolyticus* used the slide agglutination technique via the *V. parahaemolyticus* antiserum kit. The method was performed according to the manufacturer’s instructions (Denka Seiken, Tokyo, Japan). The antiserum test kit consists of 11 V*. parahaemolyticus* O and 71 K antisera. Serotyping followed the manufacturer’s instructions. Briefly, an aliquot of the bacterial cell suspension in normal saline (3% NaCl) was prepared from overnight test culture grown in TSA containing 3% NaCl. For the O serotype, the fresh prepared suspension of bacterial cells was boiled for 2 h and the boiled cells were agglutinated with specific anti-O antibodies. The remaining non-boiled bacterial cell was used for the determination of the K serotype and subjected to agglutination with specific anti-K antibodies.

### Antibiotic Resistance Pattern

The next step was according to a previously described protocol following the Kirby–Bauer disk diffusion susceptibility test [27]. An overnight culture of *V. parahaemolyticus* was suspended in 5 ml sterile aliquots of normal saline adjusted to 2% NaCl. Sterile cottonwool swabs were used for each test suspension and were inoculated onto Muller-Hinton Agar supplemented with 2% NaCl. Twenty-eight Oxoid antibiotic discs (Oxoid, England) were commercially available and represented 10 classes of antibiotics. Antibiotic disks were placed onto Muller–Hinton Agar using an automated disk dispenser (Oxoid, UK). The diameter of the zone of inhibition was measured in mm using Vernier calipers, and interpretation of the results was recorded as sensitive (S), intermediate (I), or resistant (R) based on breakpoints for *Vibrio* species according to published protocol by Hudzicki (2012) and the Clinical and Laboratory Standards Institute (CLSI). The following antibiotic agents were tested: cephalothin (KF, 30 μg), ceftazidime (CAZ, 30 μg), cefaclor (CEC, 30 μg), cefepime (FEP, 30 μg), cefpodoxime (CPD, 10 μg), cefotaxime (CTX, 30 μg), ceftriaxone (CRO, 30 μg), ceftizoxime (ZOX, 30 μg), cefixime (CFM, 5 μg), ampicillin (AMP, 10 μg), amoxycillin/clavulanic acid (AMC, 20/10 μg), carbenicillin (CAR, 100 μg), piperacillin (PRL, 100 μg), piperacillin/tazobactam (TZP, 100/10 μg), ticarcillin (TIC, 75 μg), nalidixic acid (NA, 30 μg), norfloxacin (NOR, 10 μg), levofloxacin (LEV5 μg), amikacin (AK, 30 μg), gentamicin (CN, 10 μg), neomycin (N, 30 μg), colistin (CT, 10 μg), polymyxin B (PB, 300 U), aztreonam (ATM, 30 μg), chloramphenicol (C, 30 μg), imipenem (IPM, 10 μg), trimethoprim/sulfamethoxazole (SXT, 1.25/23.75 μg), and tetracycline (TE, 30 μg). The reference strain *E.coli* ATCC25922 was used as a control while performing antimicrobial susceptibility tests. The calculation of multiple antibiotics resistance (MAR) index was performed for *V. parahaemolyticus* isolates as described elsewhere [28]. Briefly, the MAR index was calculated by applying the formula MAR = a/b, where “a” represents the antibiotic to which the test isolate showed resistance, and “b” is the total number of antibiotics. A value greater than 0.2 indicates that the isolates were isolated from high-risk sources [29].

### Molecular Subtyping Using AP-PCR

AP-PCR was performed using arbitrarily random primer AP2 (5’-GTTTCGCTCC-3’) as described previously [5, 11]. Briefly, 50 ng of extracted DNA as described above was used for AP-PCR. The PCR reaction amplification was carried out in a 25-µl mixture composed of 2 µl of the template DNA, 2.5 µl of 10 × reaction buffer (Promega, USA), 1.25 µl of 25 mM MgCl_2_, 0.5 µl of *Taq* polymerase (5 U/µl), 1.25 µl of 2.5 mM deoxynucleoside triphosphates solution, 2 µl of 25 pmol of AP-2 primer, and 15.5 µl of nuclease free water. The amplification reactions were performed using a Swift MaxPro thermocycler (ESCO, Singapore) as follows: 4 min of primarily denaturation: 95 °C, following 45 cycles of denaturation at 95 °C for 1 min, annealing fulfilled at 36 °C for 1 min, extension at 72 °C for 2 min, and the final extension at 72 °C for 7 min. Furthermore, 10 µl of the PCR-amplified products with both AP-2 primer was separated on a 1.5% agarose gel electrophoresis for 120 min at 90 Volt. A GelPiolt 1 kb Plus ladder (Qiagen, Germany) was used as a DNA molecular weight marker.

### DNA Fingerprinting Analysis

The analysis of DNA fingerprints generated by AP-PCR were processed using GelJ software to analyze the DNA fingerprint [30]. The unweighted average pair group method (UPGMA) and a Dice similarity coefficient were used to investigate the genetic relationship among *V. parahaemolyticus* isolates. The analysis of DNA fingerprints was performed at position tolerance and optimization value of 1%. Discrimination of AP fingerprints were implemented with a cut-off of 85% to differentiate the number of AP fingerprint types among the genotyped isolates of *V. parahaemolyticus*.

### Statistical Analysis

Descriptive statistics were used to summarize the distributions of temperature and salinity for the seawater parameters at the sampling sites. The Spearman’s rank correlation was used to assess the association between the two parameters. The MAR values were compared with 0.2 (as a mean value) using the one sample *t* test and compared with 0.2 again (as a median value) using the Wilcoxon test. PAST software was used for these comparisons [31].

## Results

### Water Physical Parameters

The seawater surface temperature values ranged from 17.4 °C to 32.7 °C during sample collection from April 2015 to January 2016 (Table 1). The interquartile range was 11 °C, the mean temperature was 27.2 °C, and the median temperature was 29.9 °C. The highest water temperature was documented during the month of October 2015 in the Dammam corniche. Water salinity values ranged from 29.4 to 45.8 psu/ppt. The interquartile range was 6.875 psu/ppt, the mean salinity was 41.06 psu/ppt, and the median salinity was 43.6 psu/ppt. The highest salinity was recorded during the month of October 2015 in Tarout corniche (TRC) and Fanateer corniche (FNC) during the month of April 2015 while the lowest was recorded during the month of October 2015 in Qatif corniche (QTC). There was no correlation between temperature and salinity (Spearman’s *r* = 0.08, *p* = 0.83).

### Occurrence of *V. parahaemolyticus*

Table 3 describes the samples location and number of *V. parahaemolyticus* recovered on CHROMagar Vibrio and confirmation of isolates at species level by PCR targeted to the *tox*R gene. A total of 27 (6.8%) of 400 seawater samples examined were positive and yielded 110 presumptive isolates. Of the 110 presumptive isolates of *V. parahaemolyticus*, 40 isolates were positive for the *tox*R gene as shown in Table 3.

**Table 3:** Occurrence of *V. parahaemolyticus*

Sampling site (site no.)	Sampling date	No. of samples	No. of positive samples	No. of presumptive colonies recovered on *CV agar	No. of colonies confirmed by *tox*R-PCR
FNC (1)	7 April 2015	50	5	25	6
DMC (2)	26 May 2015	60	1	3	1
DMF (3)	26 May 2015	60	5	16	5
TRC (4)	19 October 2015	40	1	3	1
SEC (5)	19 October 2015	25	1	4	1
QTC (6)	19 October 2015	25	4	15	6
PBJ (7)	28 October 2015	50	1	5	1
MOI (8)	30 December 2015	40	6	28	14
SEC (9)	24 January 2016	25	1	3	1
QTC (10)	24 January 2016	25	2	8	4
Total		400	27	110	40

*CV agar; CHROM Vibrio agar

### Screening of Potential Virulence Gene Markers

In this study, none of *V. parahaemolyticus* were reported positive for major virulence genes (*tdh* and/or *trh*) genes. Moreover, none of the isolates tested positive for the pandemic clone using the GSPCR method targeted to the *tox*RS gene (Table 4).

**Table 4:** Serotype and molecular characterization of *V. parahaemolyticus* strains (*n* = 40) isolated from the coastal water of the Eastern Province of Saudi Arabia

Strain no.	Strain code	Sampling date	Location	Virulence gene			Serotype		GS-PCR	AP-PCR genotype
				*tlh*	*tdh*	*trh*	O-antigen	K-antigen		
1	VP526	26 May 2015	DMF	+	−	−	O1	K25	−	A5
2	VP527	26 May 2015	DMF	+	−	−	O1	K25	−	A5
3	VP535	26 May 2015	DMC	+	−	−	O10	KUT	−	A4
4	VP537	26 May 2015	DMF	+	−	−	O10	KUT	−	A4
5	VP538	26 May 2015	DMF	+	−	−	O10	KUT	−	A2
6	VP539	26 May 2015	DMF	+	−	−	O10	KUT	−	A4
7	VP552	7 April 2015	FNC	+	−	−	O5	K17	−	A4
8	VP553	7 April 2015	FNC	+	−	−	O5	K17	−	A4
9	VP555	7 April 2015	FNC	+	−	−	O5	K17	−	A4
10	VP556	7 April 2015	FNC	+	−	−	O5	K17	−	A2
11	VP557	7 April 2015	FNC	+	−	−	O5	K17	−	A4
12	VP558	7 April 2015	FNC	+	−	−	O5	K17	−	A4
13	VP783	19 October 2015	QTC	+	−	−	O10	KUT	−	A4
14	VP784	19 October 2015	QTC	+	−	−	O2	K28	−	A2
15	VP785	19 October 2015	QTC	+	−	−	O5	K30	−	A3
16	VP786	19 October 2015	QTC	+	−	−	O10	KUT	−	A2
17	VP787	19 October 2015	QTC	+	−	−	O10	KUT	−	A2
18	VP788	19 October 2015	QTC	+	−	−	O10	KUT	−	A2
19	VP791	19 October 2015	SEC	+	−	−	O10	KUT	−	A4
20	VP796	19 October 2015	TRC	+	−	−	O10	KUT	−	A1
21	VP805	28 October 2015	PBJ	+	−	−	O1	KUT	−	A2
22	VP834	30 December 2015	MOI	+	−	−	O10	KUT	−	A1
23	VP835	30 December 2015	MOI	+	−	−	O10	KUT	−	A3
24	VP838	30 December 2015	MOI	+	−	−	O10	KUT	−	A1
25	VP839	30 December 2015	MOI	+	−	−	O10	KUT	−	A4
26	VP840	30 December 2015	MOI	+	−	−	O10	KUT	−	A3
27	VP841	30 December 2015	MOI	+	−	−	O10	KUT	−	A3
28	VP842	30 December 2015	MOI	+	−	−	O10	KUT	−	A3
29	VP843	30 December 2015	MOI	+	−	−	O10	KUT	−	A3
30	VP844	30 December 2015	MOI	+	−	−	O10	KUT	−	A3
31	VP845	30 December 2015	MOI	+	−	−	O10	KUT	−	A3
32	VP846	30 December 2015	MOI	+	−	−	O10	KUT	−	A2
33	VP847	30 December 2015	MOI	+	−	−	O10	KUT	−	A2
34	VP848	30 December 2015	MOI	+	−	−	O10	KUT	−	A3
35	VP849	30 December 2015	MOI	+	−	−	O10	KUT	−	A2
36	VP850	24 January 2016	QTC	+	−	−	O10	KUT	−	A3
37	VP851	24 January 2016	QTC	+	−	−	O5	K30	−	A3
38	VP852	24 January 2016	QTC	+	−	−	O5	K30	−	A3
39	VP853	24 January 2016	QTC	+	−	−	O5	K30	−	A3
40	VP856	24 January 2016	SEC	+	−	−	O10	KUT	−	A2

### Serotyping of *V. parahaemolyticus*

**Fig. 2:**
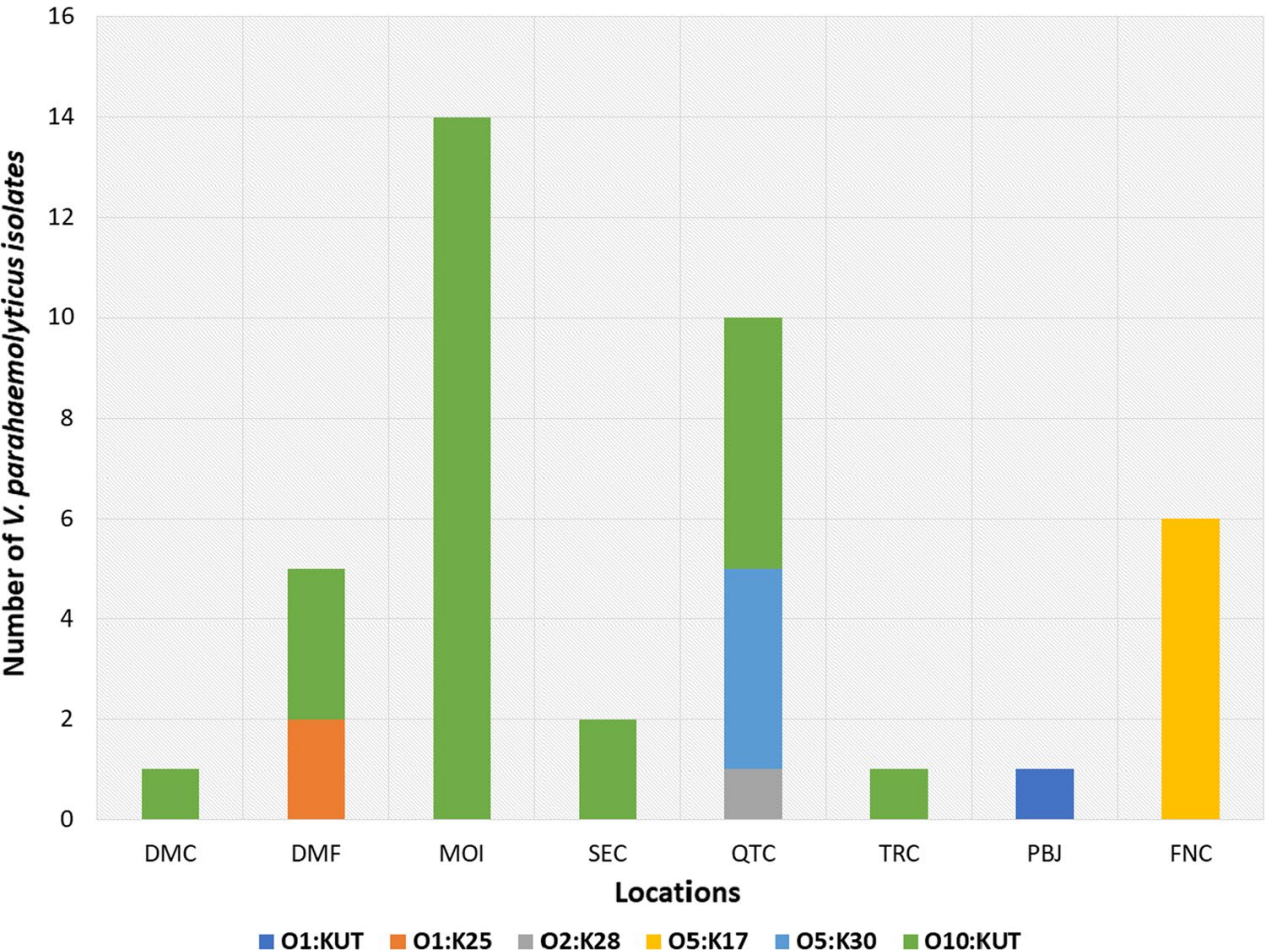
Serovar distribution of *V. parahemolyticus* isolated from coastal areas in Eastern Province, Saudi Arabia

**Fig. 3:**
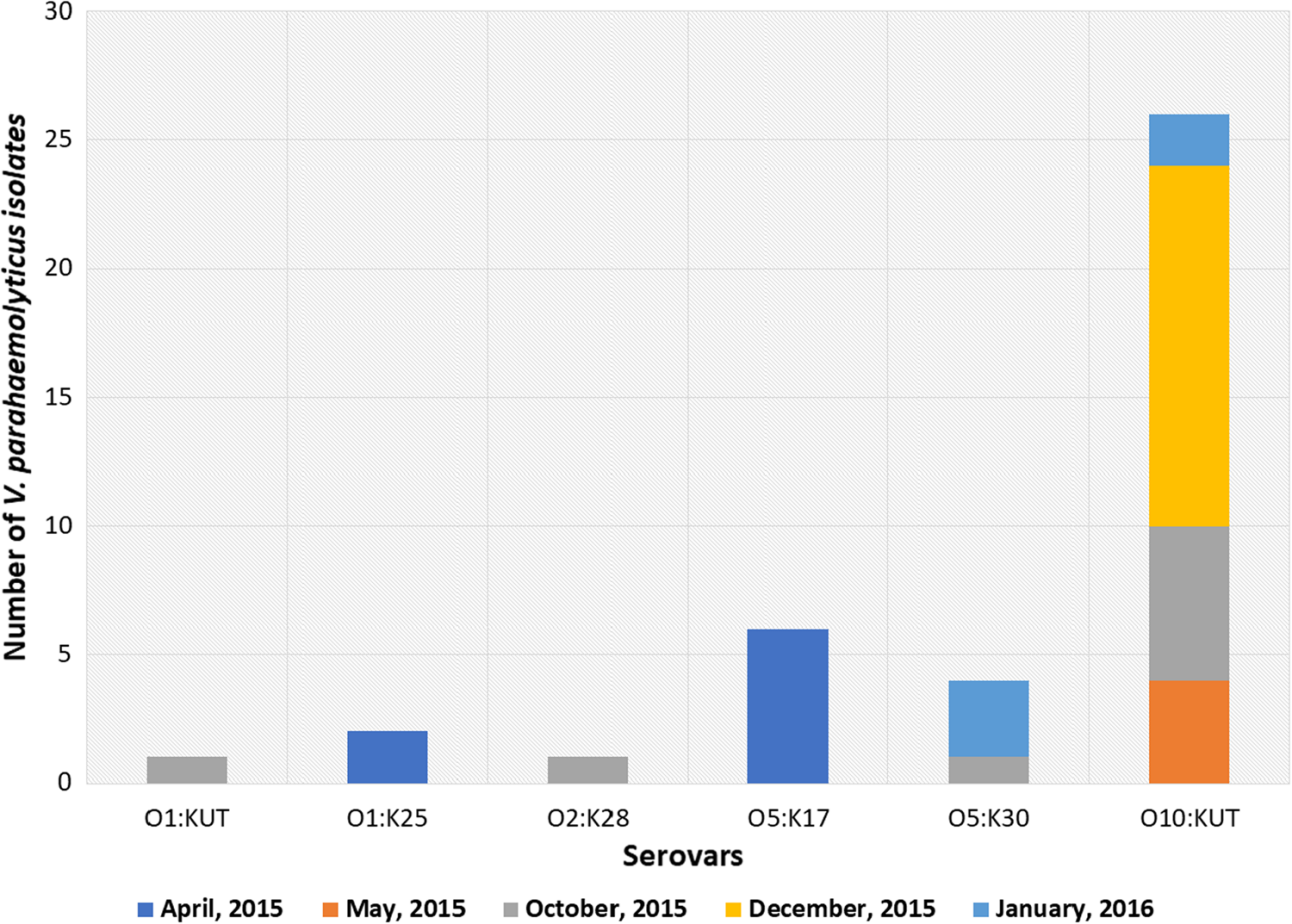
Monthly serovars distribution of *V. parahemolyticus* isolated from coastal areas in Eastern Province, Saudi Arabia

For epidemiological purposes, serotyping was performed on all 40 isolates using O and K antisera. The distribution of *V. parahaemolyticus* serovars according to sample locations is presented in Fig. 2. Thirteen (32.5%) defined serotypes were identified and 27 (67.5%) isolates underwent O antisera serotyping; however, they did not react with K antisera and resulted in untypeable (UT) serovars (Table 4). The most prevalent serovar reported in this study was O10:KUT with 26 isolates (Fig. 2), six isolates were O5:K17, four isolates were O5:K30, two isolates were O1:K25 and the least prevalent were single isolates from O1:KUT and O2:K28, respectively. O1:K25 and O5:K17 serovars were recovered from seawater samples collected during the month of April 2015, whereas O1:KUT and O2:K28 were isolated from seawater samples collected during the month of October 2015 (Fig. 3). One and three isolates of O5:K30 serovar were recovered only from QTC locations during October 2015 and January 2016, respectively (Fig. 3). Furthermore, the O10:KUT serovar was isolated over all months of sampling except April 2015. This was recovered from all sampling locations except FNC and PBJ sites (Figs. 2 and 3).

### Antibiotic Resistance Pattern

All isolates of *V. parahaemolyticus* were susceptible to piperacillin/tazobactam, nalidixic acid, norfloxacin, levofloxacin, neomycin, imipenem, trimethoprim/sulfamethoxazol and tetracycline (Table 5). Overall, the isolates were resistant to carbenicillin 39 (97.5%), cephalothin 26 (65%), cefaclor 21 (52.5%), colistin 19 (47.5%), and cefixime 16 (40%); 14 (35%) and 32 (80%) isolates displayed intermediate resistance to ampicillin and ticarcillin, respectively (Table 5). Regarding *V. parahaemolyticus* resistance, 35 patterns were observed. Thirty (75%) of the isolates were multiple drug resistant (MDR) with resistance to three antibiotics or more (Table 6). The high prevalence of MDR with resistance to ≥ 5 antibiotics pattern was found among 1, 2, and 18 isolates of O5:K17, O1:K25, and O10:KUT serovars, respectively (Table 6). Four isolates of O10:KUT serovars (VP850, VP796, VP845, and VP849) isolated from QTC, TRC, and MOI sites during different sampling periods were found with similar resistance patterns (Table 6). The single isolate of O1:KUT serogroup isolated from PBJ site in this study was found to be resistant to cefixime and susceptible to all analyzed antibiotic classes. Similarly, a lower resistance was found among two (VP553 and VP556), three (VP785, VP851 and VP852), and four isolates (VP535, VP788, VP834 and VP856) of O5:K17, O5:K30 and O10:KUT serovars, respectively. These exhibited resistance to one or two antibiotic classes (Table 6). The MAR index values were recorded in the range from 0.03 to 0.37. However, the mean and median MAR index are significantly lower than the cut-off value of 0.2 (median = 0.18 and mean = 0.16). The antibiotic resistance patterns of isolates revealed that 12 (30%) of *V. parahaemolyticus* isolates recorded very significant MAR indexes above the range of 0.2 (Table 6). Eight isolates of O10:KUT serovar isolated from MOI site expressed the highest MAR indices among all sites investigated here. The resistance patterns with highest MAR values above 0.3 indices occurred among two strains of O1:K25 (VP527) and O10:KUT (VP835) serovars isolated from DMF and MOI, respectively (Table 6).

**Table 5:** Antibiotic susceptibility testing of *V. parahaemolyticus* isolated from coastal water (*n* = 40)

Antibiotic class	Antibiotic	Drug content (µg)	S (%)	I (%)	R (%)
Cephalosporins	Cephalothin (KF)	30	3 (7.5)	11 (27.5)	26 (65)
	Ceftazidime (CAZ)	30	34 (85)	0	6 (15)
	Cefaclor (CEC)	30	14 (35)	5 (12.5)	21 (52.5)
	Cefepime (FEP)	30	37 (92.5)	0	3 (7.5)
	Cefpodoxime (CPD)	10	18 (45)	11 (27.5)	11 (27.5)
	Cefotaxime (CTX)	30	38 (95)	0	2 (5)
	Ceftriaxone (CRO)	30	39 (97.5)	1 (2.5)	0
	Ceftizoxime (ZOX)	30	38 (95)	1 (2.5)	1 (2.5)
	Cefixime (CFM)	5	15 (37.5)	9 (22.5)	16 (40)
Penicillins	Ampicillin (AMP)	10	22 (55)	14 (35)	4 (10)
	Amoxycillin/clavulanic acid (AMC)	20/10	30 (75)	2 (5)	8 (20)
	Carbenicillin (CAR)	100	0	1 (2.5)	39 (97.5)
	Piperacillin (PRL)	100	39 (97.5)	0	1 (2.5)
	Piperacillin/tazobactam (TZP)	100/10	40 (100)	0	0
	Ticarcillin (TIC)	75	4 (10)	32 (80)	4 (10)
Quinolones	Nalidixic acid (NA)	30	40 (100)	0	0
	Norfloxacin (NOR)	10	40 (100)	0	0
	Levofloxacin (LEV)	5	40 (100)	0	0
Aminoglycosides	Amikacin (AK)	30	34 (85)	2 (5)	4 (10)
	Gentamicin (CN)	10	39 (97.5)	0	1 (2.5)
	Neomycin (N)	30	40 (100)	0	0
Polymyxin	Colistin (CT)	10	18 (45)	3 (7.5)	19 (47.5)
	Polymyxin B (PB)	300 U	31 (77.5)	5 (12.5)	4 (10)
Monobactams	Aztreonam (ATM)	30	29 (72.5)	6 (15)	5 (12.5)
Phenicols	Chloramphenicol (C)	30	35 (87.5)	0	5 (12.5)
Carbapenems	Imipenem (IPM)	10	40 (100)	0	0
Sulfonamides	Trimethoprim/sulfamethoxazole (SXT)	1.25/23.75	40 (100)	0	0
Tetracycline	Tetracycline (TE)	30	40 (100)	0	0

**Table 6:** Frequency of multidrug-resistance patterns among *V. parahaemolyticus* isolates

Strain no.	Strain code	Sample location	Resistance patterns	MAR index
15	VP785	Qatif corniche (QTC)	CAR	0.03
37	VP851	Qatif corniche (QTC)	CAR	0.03
21	VP805	Palm Beach Jubail (PBJ)	CFM	0.03
10	VP556	Fanateer corniche (FNC)	C, CAR	0.03
3	VP535	Dammam corniche (DMC)	KF, CAR	0.07
40	VP856	Sayhat corniche (SEC)	KF, CAR	0.07
8	VP553	Fanateer corniche (FNC)	CT, CAR	0.07
38	VP852	Qatif corniche (QTC)	CAR, CPD	0.07
22	VP834	Almorjan Island (MOI)	CEC, CAR	0.07
18	VP788	Qatif corniche (QTC)	AMC, CAR	0.07
12	VP558	Fanateer corniche (FNC)	KF, CAR, TIC	0.11
29	VP843	Almorjan Island (MOI)	KF, CAR, CPD	0.11
14	VP784	Qatif corniche (QTC)	AMC, CAR, PB	0.11
33	VP847	Almorjan Island (MOI)	AMC, KF, CAR	0.11
9	VP555	Fanateer corniche (FNC)	CAZ, CAR, CPD	0.11
16	VP786	Qatif corniche (QTC)	ATM, CAR, CPD	0.11
6	VP539	Dammam marina front (DMF)	KF, CEC, CT, CAR	0.14
39	VP853	Qatif corniche (QTC)	KF, CAR, CPD, CTX	0.14
7	VP552	Fanateer corniche (FNC)	CEC, FEP, CAR, CFM	0.14
1	VP526	Dammam marina front (DMF)	KF, CT, CAR, PB, TIC	0.18
11	VP557	Fanateer corniche (FNC)	C, CAZ, CAR, CPD, PB	0.18
19	VP791	Sayhat corniche (SEC)	KF, CEC, CT, FEP, CAR	0.18
36	VP850	Qatif corniche (QTC)	KF, CEC, CL, CAR, CFM	0.18
20	VP796	Tarout corniche (TRC)	KF, CEC, CT, CAR, CFM	0.18
31	VP845	Almorjan Island (MOI)	KF, CEC, CT, CAR, CFM	0.18
35	VP849	Almorjan Island (MOI)	KF, CEC, CT, CAR, CFM	0.18
30	VP844	Almorjan Island (MOI)	AMC, KF, CEC, CAR, CPD	0.18
13	VP783	Qatif corniche (QTC)	AMC, CAZ, CEC, CAR, TIC	0.18
34	VP848	Almorjan Island (MOI)	ATM, C, KF, CEC, CT, CAR	0.21
26	VP 40	Almorjan Island (MOI)	KF, CEC, CT, CAR, CPD, CFM	0.21
5	VP538	Dammam marina front (DMF)	AMP, KF, CEC, CT, CAR, CFM	0.21
25	VP839	Almorjan Island (MOI)	KF, CEC, CT, CAR, CPD, CFM	0.21
4	VP537	Dammam marina front (DMF)	C, KF, CAZ, CEC, CT, CAR, CPD	0.25
28	VP842	Almorjan Island (MOI)	ATM, AK, KF, CT, FEP, CAR, CFM	0.25
27	VP841	Almorjan Island (MOI)	AMC, KF, CEC, CAR, CFM, CN, PRL	0.25
17	VP787	Qatif corniche (QTC)	AK, KF, CEC, CT, CAR, CPD, ZOX, CFM	0.29
24	VP838	Almorjan Island (MOI)	AMP, AMC, AK, KF, CEC, CT, CAR, CFM	0.29
32	VP846	Almorjan Island (MOI)	AMP, ATM, AK, KF, CEC, CT, CAR, CFM	0.29
2	VP527	Dammam marina front (DMF)	AMP, KF, CAZ, CEC, C, CAR, CFM, PB, TIC	0.32
23	VP835	Almorjan Island (MOI)	AMC, ATM, C, KF, CAZ, CEC, CT, CAR, CTX, CFM	0.37

### Molecular Subtyping Using AP-PCR

**Fig. 4:**
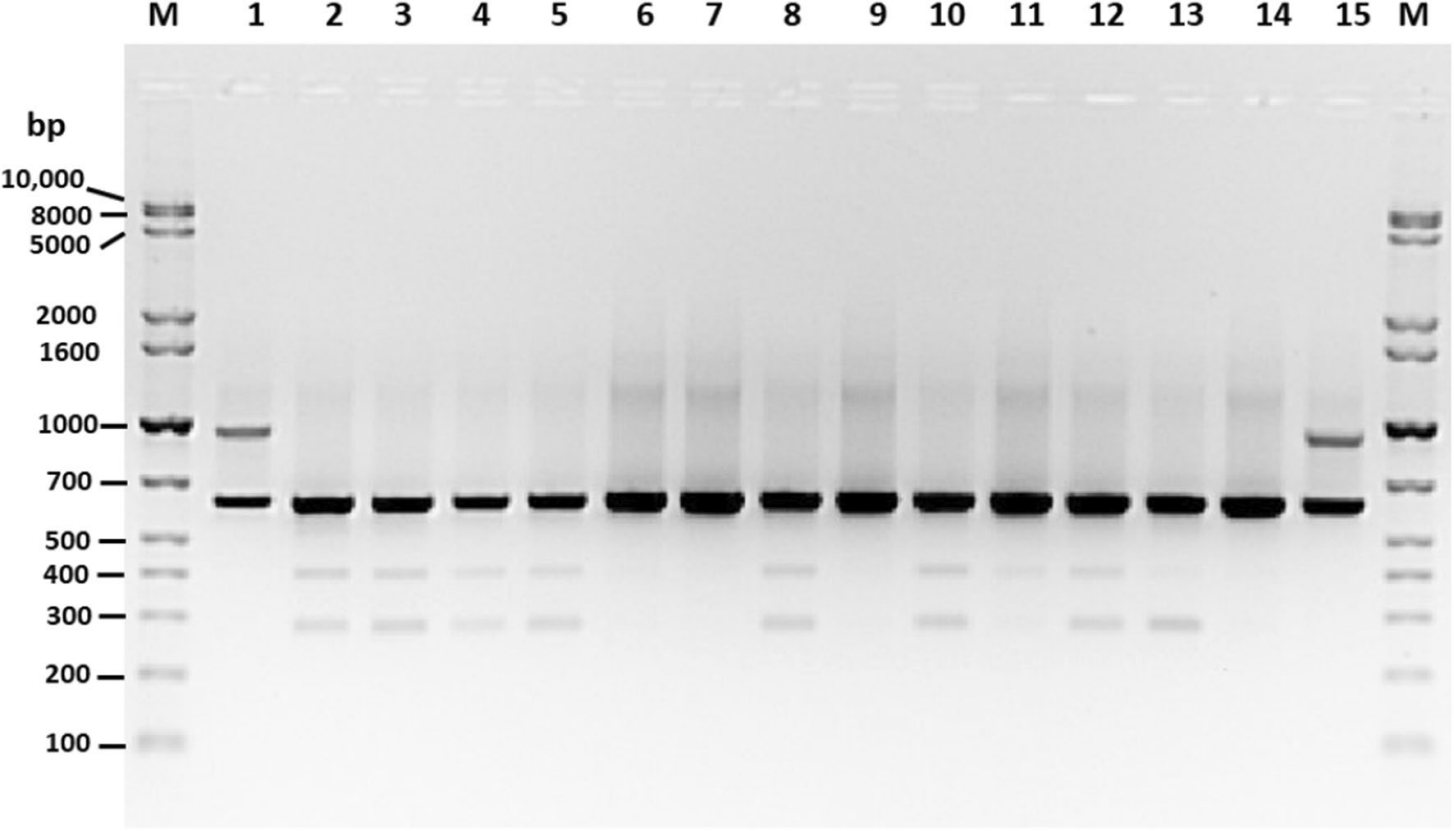
Representative AP-PCR fingerprints of *V. parahaemolyticus*. M; GelPiolt 1 kb Plus ladder

**Fig. 5:**
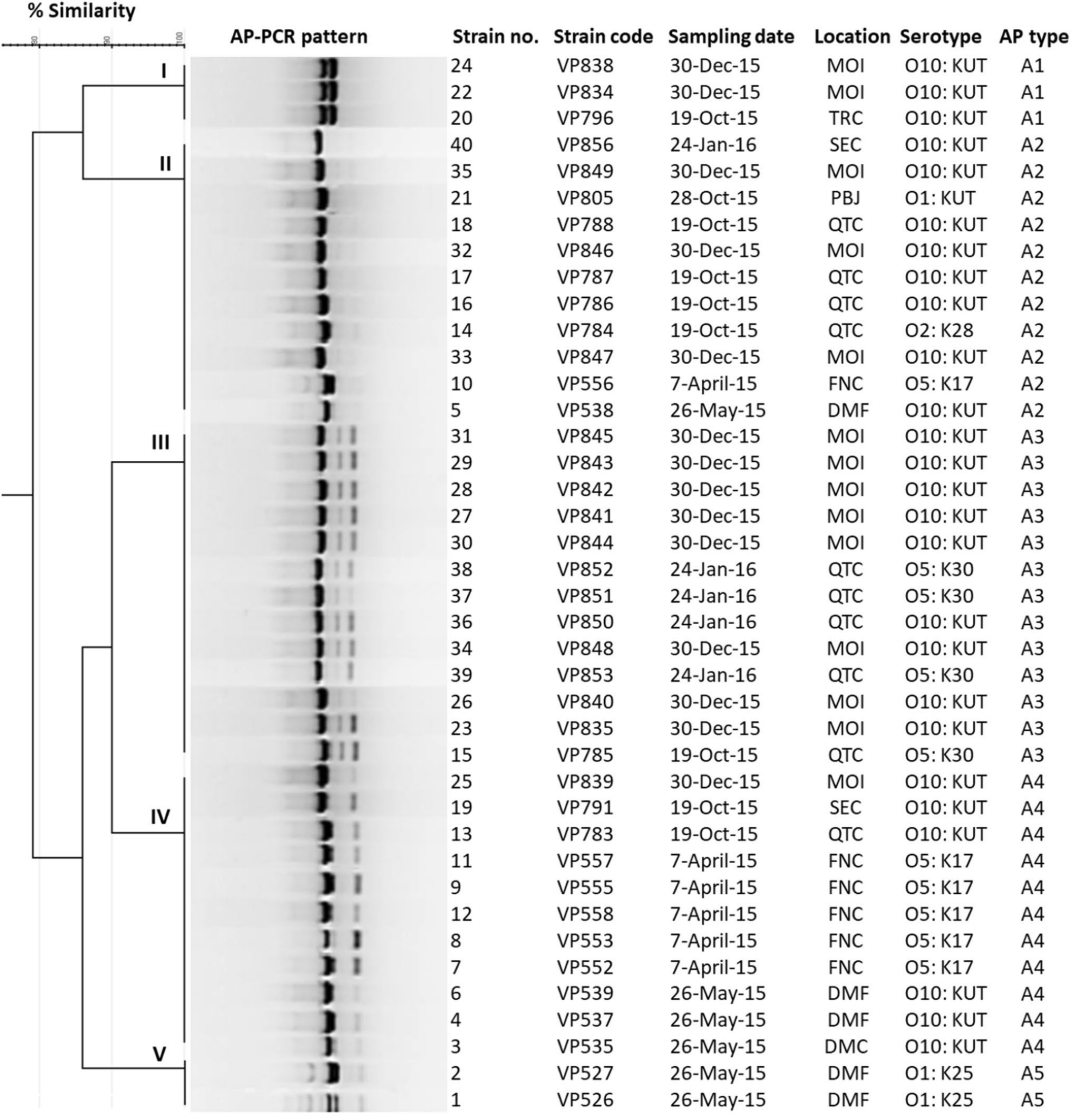
Clusters of UPGMA dendrogram analysis using AP-PCR fingerprinting

AP-PCR could type all 40 isolates investigated here. The fingerprints were achieved using the AP-2 arbitrarily primer sequence, which revealed 3 to 5 bands ranging in size from 300 to 1000 bp and approximately 670 bp DNA fragments. This was shared between electrophoresed AP-PCR amplicons for all isolates (Fig. 4). The AP-PCR fingerprints grouped the 40 isolates into five clusters (I to V) at a cut-off value of 85% to assign the AP fingerprint types (A1, A2, A3, A4, and A5) as presented in Table 4 and Fig. 5. Among all clusters, cluster III (A3) had the largest number of isolates and comprised 4 and 9 isolates belonging to serovar O5:K30 and O10:KUT; these were isolated from QTC and MOI sites, respectively (Fig. 5). Four isolates (VP785, VP851, VP852, and VP853) of O5:K30 serovar isolated from QTC location during October 2015 and January 2016 were grouped together in cluster III (Fig. 5). Similarly, five of six isolates of O5:K17 serovar isolated from FNC site during April 2015 were grouped together in cluster IV with nine isolates of O10:KUT serovars recovered from different geographical sites and time intervals (Fig. 5).

## Discussion

*V. parahaemolyticus* is one of the leading causes of foodborne infection especially after ingestion of contaminated seafood [32]. Here, we examined seawater samples collected from different sites along the coastal areas of the Arabian Gulf in the Eastern Province of Saudi Arabia. Of the 400 seawater samples examined here, *V. parahaemolyticus* was recovered from 27 samples. The distribution of *V. parahaemolyticus* in the coastal water environment has been documented in different coastal areas in the world and was isolated from the seawater surface and sediment. The presence and survival of this bacterium in the marine environment are related and connected to environmental parameters such as sea surface temperature and salinity [33–35]. The overall isolation rate of *V. parahaemolyticus* was 6.8% in the coastal water. The highest number of positive water samples for *V. parahaemolyticus* was collected from MOI and the recorded water salinity and surface water temperature during the month of December 2015 was 43.6 ppt and 17.4 °C, respectively (Table 1).

The spread and epidemiology of infections are related to water temperatures, and most outbreaks of *V. parahaemolyticus* occur during the warmer months. The reported appropriate temperature for the proliferation of *V. parahaemolyticus* is 30 °C [37]. Recently, several reports from new coastal areas worldwide have documented the occurrence of *V. parahaemolyticus*; these incidences are related to an increase in seawater temperatures [5, 10, 38–40]. This climatic change in temperature is responsible for outbreaks of diarrhea caused by *V. parahaemolyticus* in places such as Alaska where seawater temperatures are normally low and *V. parahaemolyticus* infection is very rare [41]. Outbreaks were also seen in southern Chile at Puerto Montt between 2004 and 2007. However, in this study we found that all 40 isolates of *V. parahaemolyticus* strains tested negative for *tdh* and *trh* genes and no isolates tested positive for the pandemic clone of *V. parahaemolyticus* O3:K6 while using group-specific PCR (GS-PCR). Strains of *V. parahaemolyticus* harboring *tdh* or *trh* genes can cause gastroenteritis in humans [6]. Our study is consistent with several studies showing that environmental strains rarely possess the *tdh* and/or *trh* virulence genes [1, 6, 26, 42, 43]. However, the presence of these were found in up to 90% of clinical strains of *V. parahaemolyticus* [5, 44, 45].

Recent studies have shown that from 1996 to date, at least 21 different serotypes of *V. parahaemolyticus* appeared to have identical genotypes and molecular profiles to those of O3:K6; these were collectively described as “serovariants” of O3:K6 isolates in which the widespread serotypes were O4:K68, O1:K25, and O1:KUT “untypeable” [1, 11]. A possible theory is divergence of these serotypes from the O3:K6 isolates by alteration of O and/or K antigens [46]. A recent study has suggested that the O/K antigen modification is a biological characteristic of the pandemic *V. parahaemolyticus* needed for survival in the face of host immunological resistance and varying external environments [47]. In our present study, we detected three serotypes (O1: KUT, O1:K25 and O5:K17) that were included in the pandemic group of O3:K6 clonal serovariants; all of these lacked genetic markers of the pandemic clone (*tdh*^−^/*tox*RS^−^) (Table 4). In general, an isolate of *V. parahaemolyticus* will not be considered to be a pandemic clone unless it harbors *tdh* and *tox*RS (*tdh*^+^/*tox*RS^+^) [48].

Recently, a pandemic clone carrying *tdh*^+^ and *tox*RS^+^ was reported in Europe, the United States, Mexico, Bangladesh, and China [8, 49]. However, our current findings contradict these studies. Our findings agree with the concerns raised by Jones and colleagues regarding the reliability of the *tdh* and *trh* genes as virulence markers—their studies reported negative strains for *tdh* and *trh* [50]. Furthermore, the same study indicated that both *tdh*, *trh*, and T3SS2 genes are not necessarily predictive of pathogenic potential; their study highlighted the need for more-detailed pathogenicity investigations of *V. parahaemolyticus* [50]. A recent study from India investigated the prevalence of *V. parahaemolyticus* in retail seafood in Kerala. That study reported that none of the isolates harbored *tox*RS although 14 out of 29 isolates were positive for the *tdh* gene [51].

A study conducted in southern Thailand investigated 865 clinical strains of *V. parahaemolyticus* isolated from patients at Hat Yai hospitals between 2000 and 2005; the study results revealed that there was a significant decrease in the proportion of infections by pandemic strain of *V. parahaemolyticus* [52]. Therefore, all strains isolated from patients from 2003 to 2004 were obtained after screening for major virulence gene markers, the O:K serotype, and pandemic clones using GSPCR defined as GSPCR-positive *tdh*^+^*trh*^−^. These remained stable at percentages of 64.1, 67.5, 69.7, and 67.7% of the total isolates each year. In 2004 to 2005, there was a decrease of pandemic clone (GSPCR^+^*tdh*^+^*trh*^−^) percentages from 56.1 to 55.5%, respectively [52]. Similarly, two studies conducted in the southern region of Thailand reported variability properties that remained consistent with the *V. parahaemolyticus* strains isolated from individual patients; these data indicated that some patients were infected with unique strains of *V. parahaemolyticus* (GSPCR^+^*tdh*^−^*trh*^−^) suggesting that in vivo changes might have occurred in certain individuals leading to a deleted *tdh* gene [53, 54].

A study from Italy analyzed two *V. parahaemolyticus* strains isolated in May 2007 from Northern Italy seawater and plankton samples. The results showed the presence of the virulence genes *tdh* and orf8 as well as pandemic-specific markers. Interestingly, the two strains showed serotypes not included in the ‘pandemic group’ [34]. From the literature, not all strains of *V. parahaemolyticus* have the same pathogenic potential, but infections in humans are usually caused by diverse serotypes. To date, 21 serotypes are known as ‘pandemic group’ or serovariants of O3:K6 isolates based on nearly identical genotypes and molecular profiles [1]. However, as documented in literature, there is no complete agreement on a single marker for identification of pandemic clone. Such identification has been based on detection of *tox*RS gene using GSPCR and the presence of *tdh* gene (*tox*RS^+^/*tdh*^+^) [34, 48, 55]. Several reports on the pandemic clone of O3:K6 have emerged after genetic elements were transferred to the pathogenic strains to increase their robustness and ability to cause infection in human [56, 57]. Here, positive strains for *tox*RS were not detected, but constant surveillance is highlighted to detect the emergence of any pandemic clones. Of note, comparatively clinical strains were reported and did not contain virulence gene markers of *tdh* and *trh* [4]. While most hemolysins were absent, *V. parahaemolyticus* remained as toxigenic and elucidated the expression of other virulence activities [58–61].

Recent studies have indicated the detection of MDR among strains of *V. parahaemolyticus* isolated from coastal water and fisheries products versus clinical isolates [29, 62–66]. Here, 60% of *V. parahaemolyticus* isolates were MDR (Table 6). Our study is consistent with other reports showing that *V. parahaemolyticus* is increasingly resistant towards cephalosporins and penicillins [66–68]. In this study, the antibiotic resistance patterns among *V. parahaemolyticus* indicated that 12 (30%) of isolates had significant MAR index values above 0.2, while the highest MAR index value above 0.3 indices occurred among two strains of O1:K25 and O10:KUT serovars isolated from DMF and MOI sites, respectively. This agrees with other studies elsewhere that detected an MAR index above 0.2 among 45% of pandemic and non-pandemic *V. parahaemolyticus* isolated from seafood [69]. The MAR indices that exceed 0.2 indicated that these isolates originated from high-risk contamination and may pose human risks [70, 71].

In the case of *V. parahaemolyticus*, this may be due to overuse of antibiotics by fisheries and aquaculture [72]. Although the availability of those antibiotics is important for the productivity and food security, their inappropriate use undermines their benefits [73]. Recently, the FAO of the United Nations established an action plan to improve awareness of antimicrobial resistance to prevent the excessive use of antibiotics (FAO, 2016). In addition, seawater is becoming contaminated by disposal of medical waste as well as sewage—these all contain antibiotics from human and animals. These can exacerbate resistance in pathogenic bacteria [72]. The transmission of multidrug resistance genes can undermine the antibiotic used for treatment of vibrio infection. The resistance gene might complicate the treatment of severe vibriosis infections [74, 75]. Moreover, the presence of drug resistance genes in marine environments can lead to potential reservoirs that might play role in transferring these resistance genes to pathogenic bacteria through horizontal gene transfer via conjunction, transformation, or transduction [63]. Consequently, the environment has a remarkable role in the global spread of the clinically relevant antibiotic resistance and therefore imposes significant human health risks [76].

The analysis of AP-PCR fingerprints revealed that there is no site or location that influences the clustering of the isolates except for two isolates of serotype O1:K25 isolated from DMF; these were found in a single cluster and exhibited an identical pattern (Fig. 5). Also, six isolates of serotype O5:K17 recovered from FNC location were clustered with other isolates recovered from different sampling sources. Our findings are consistent with a recent study from Kerala in India, which investigated genetic relatedness on *V. parahaemolyticus* seafood isolates. This study revealed that the impact of geographical factors could be excluded because all the isolates were collected from a single location and did not group into one cluster [51]. Interestingly, we observed that all isolates genotyped by AP-PCR shared approximately 670 bp DNA fragment between electrophoresed AP-PCR amplicons thus suggesting that these isolates are clonally related. The results of the AP-PCR analysis were also consistent—the results support the view of other AP-PCR experiments that those pandemic serotypes and other emerged serovars show almost identical fragment patterns suggesting that these strains in the pandemic group might have originated from the same clone [5, 11, 48, 77]. The AP-PCR method is frequently used to investigate the genetic relationship among *V. parahaemolyticus* strains isolated from different sources including clinical samples [23, 48, 53].

## Conclusion

This study reported the first investigation and detection of *V. parahaemolyticus* O3:K6 pandemic clone serovariants of O1: KUT, O1:K25, and O5:K17 included within the ‘pandemic group’ recovered from the coastal environment in Saudi Arabia. The isolation of *V. parahaemolyticus* serotypes within the ‘pandemic group’ in the marine environment might constitute a public health concern if consumed in contaminated seafood. However, the genetic pandemic marker and virulence genes are usually associated with clinical strains isolated from stool specimens and, to date, these are hardly detected in seafood and environmental samples. The isolation of *V. parahaemolyticus* serovariants occurs in the marine environment, and none of the isolates positive for *tdh* and *trh* can pose a public health concern. Our study along with a similar study conducted in the Georgian coast of the Black Sea—found that none of *V. parahaemolyticus* isolates were positive for *tdh* and *trh* genes [39]. This study demonstrated the global spread and dissemination of *V. parahaemolyticus* serovariants in the marine environment. Its presence in the coastal environment in the Eastern Province of Saudi Arabia requires long-term monitoring consideration.

## Data Availability

The authors confirm that the data supporting the findings of this study are available within the article.

## Abbreviations

GCC: Gulf Cooperation Council
PCR: Polymerase chain reaction
AP-PCR: Arbitrarily primed-polymerase chain reaction
MAR: Multiple antibiotic resistance
GPS: Global positioning system
UT: Un-typeable
TDH: Thermostable direct hemolysin
TRH: Thermostable-related hemolysin
GS-PCR: Group-specific-polymerase chain reaction
FNC: Fanateer Corniche
DMC: Dammam Corniche
DMF: Dammam Marina Front
TRC: Tarout Corniche
SEC: Sayhat Corniche
QTC: Qatif Corniche
PBJ: Palm Beach Jubail
MOI: Almorjan Island
APW: Alkaline peptone water
NaCl: Sodium chloride
TSB: Tryptic soy broth
ATCC: American Type Culture Collection
LB: Luria–Bertani
CV: CHROM Vibrio
CLSI: Clinical and Laboratory Standards Institute
KF: Cephalothin
CAZ: Ceftazidime
CEC: Cefaclor
FEP: Cefepime
CPD: Cefpodoxime
CTX: Cefotaxime
CRO: Ceftriaxone
ZOX: Ceftizoxime
CFM: Cefixime
AMP: Ampicillin
AMC: Amoxycillin/clavulanic acid
CAR: Carbenicillin
PRL: Piperacillin
TZP: Piperacillin/tazobactam
TIC: Ticarcillin
NA: Nalidixic acid
NOR: Norfloxacin
LEV5: Levofloxacin
AK: Amikacin
CN: Gentamicin
N: Neomycin
CT: Colistin
PB: Polymyxin B
ATM: Aztreonam
C: Chloramphenicol
IPM: Imipenem
SXT: Trimethoprim/sulfamethoxazole
TE: Tetracycline
UPGMA: Unweighted average pair group method
PAST: Paleontological statistics
T3SS: Type III secretion system
MDR: Multiple drug resistance
